# EXERCISE AND IMMUNE FUNCTION FROM GESTALT COHORT: THE BENEFICIAL EMERGING ROLE OF INTEGRATED STRESS RESPONSE

**DOI:** 10.1093/geroni/igae098.2624

**Published:** 2024-12-31

**Authors:** Stefano Donega, Nirad Banskota, Amit Singh, Mary Kaileh, Arsun Bektas, Supriyo De, Ranjan Sen, Luigi Ferrucci

**Affiliations:** National Institute on Aging, Baltimore, Maryland, United States; National Institutes of Health, Bethesda, Maryland, United State; National Institute on Aging, Baltimore, Maryland, United States; National Institutes of Health, Bethesda, Maryland, United State; National Institute on Aging, Baltimore, Maryland, United States; National Institute on Aging, Baltimore, Maryland, United States; National Institute on Aging, Baltimore, Maryland, United States; National Institute on Aging, Baltimore, Maryland, United States

## Abstract

Exercise significantly impacts immune function by influencing physiological health, which in turn reduces disease susceptibility, combats immune-senescence, and lowers infection risks. Despite these effects, there are still important unanswered questions regarding the impact of exercise on the transcriptome of immune cells, age-related responses, inflammation modulation, and anti-aging benefits. In the Genetic and Epigenetic Signatures of Translational Aging Laboratory Testing (GESTALT) study involving 99 participants aged 22-92, RNA-sequencing was conducted in parallel on 11 immune cell types - B cells, T/CD4+, T/CD8+ (naïve and effector/central memory) cells, monocytes, granulocytes, and natural killer cells - to investigate their correlation with exercise (VO2 peak) using Differential Gene Expression (DGE) and Gene Set Enrichment Analysis (GSEA). Lymphoid B, T and NK cells, exhibited distinct clustering from myeloid cells in both young and old participants (< 44 and >66 years old), with 26 shared genes identified across multiple immune-cell types, reflecting enrichment for 14 energy-mitochondrial pathways associated with increased exercise. Interestingly, while mRNA splicing pathways were upregulated in the young group, older individuals displayed upregulated pathways linked to the Integrated Stress Response (ISR), a signaling pathway previously found to be activated by ER stress, hypoxia, amino acid deprivation, glucose deprivation, and viral infection etc. By utilizing a scoring system for inflammation, senescence, and mitochondria (QuickGO-EBI, SenMayo, and MitoCarta 3.0), we identified clusters of shared genes affected by physical activity. Overall, the findings suggest that stress-induced molecular responses to enhanced aerobic capacity and exercise offer potential benefits for improving the health and well-being of older individuals.

